# Angiosarcoma of the Scalp and Face: A Dosimetric Comparison of HDR Surface Applicator Brachytherapy and VMAT

**DOI:** 10.1155/2020/7615248

**Published:** 2020-08-25

**Authors:** Devarati Mitra, Yaguang Pei, Ivan Buzurovic, Phillip M. Devlin, Katherine Thornton, Chandrajit P. Raut, Elizabeth H. Baldini, Miranda B. Lam

**Affiliations:** ^1^Department of Radiation Oncology, Brigham and Women's Hospital and Dana-Farber Cancer Institute, Boston, MA, USA; ^2^Division of Radiation Oncology, MD Anderson Cancer Center, Houston, TX, USA; ^3^Department of Medical Oncology, Brigham and Women's Hospital and Dana-Farber Cancer Institute, Boston, MA, USA; ^4^Sarcoma and Bone Cancer Treatment Center, Dana-Farber Cancer Institute, Boston, USA; ^5^Department of Surgery, Brigham and Women's Hospital and Dana-Farber Cancer Institute, Boston, MA, USA

## Abstract

**Purpose:**

Angiosarcoma of the face and scalp is a rare disease with high rates of recurrence. The optimal treatment approach is not well defined. This study presents a dosimetric comparison of high-dose-rate surface applicator (HDR-SA) brachytherapy to volumetric-modulated arc therapy (VMAT).

**Methods:**

Between 2011 and 2018, 12 patients with primary or recurrent angiosarcoma of the face or scalp were treated with HDR-SA brachytherapy using CT-based planning at our institution. For comparison, deliverable VMAT plans for each patient were generated, and dose distribution was compared to the delivered HDR-SA brachytherapy plans.

**Results:**

Both VMAT and HDR-SA brachytherapy plans delivered good coverage of the clinical target. However, the dose distribution of VMAT was significantly different from HDR-SA brachytherapy across a variety of parameters. Mean doses to the lacrimal gland, orbit, lens, and cochlea were significantly higher with HDR-SA brachytherapy vs. VMAT. Brain Dmax, V80%, and V50% were also significantly higher with HDR-SA brachytherapy.

**Conclusions:**

There may be dosimetric advantages to VMAT over HDR-SA brachytherapy for many patients. However, individual tumor location, patient anatomy, and treatment reproducibility may result in HDR-SA brachytherapy being the preferred technique in a subset of patients. Ultimately, a personalized approach is likely to be the optimal treatment plan.

## 1. Introduction

Angiosarcoma is a rare malignant tumor of the vascular endothelium which accounts for 5% of cutaneous soft tissue sarcomas [1]. About 60% of these cases occur in the head and neck area, most commonly the scalp and face [2]. Although upfront surgical resection is a potential treatment approach, several studies have shown a high local recurrence rate following wide excision, and these recurrences are often associated with high morbidity [3–7]. Also, for many patients, the disease is too diffuse for upfront resection.

Radiation techniques for face and scalp angiosarcoma have evolved over time from mixed lateral photon and electron approaches to more modern external beam radiotherapy techniques or high-dose rate surface applicator (HDR-SA) brachytherapy [8–13]. Our institutional practice has been to treat angiosarcoma of the face and scalp with HDR-SA brachytherapy, but whether this is the optimal approach for the majority of patients is not well characterized. While intensity-modulated radiotherapy (IMRT) and HDR-SA brachytherapy have been dosimetrically compared in a case report, given recent data suggesting volumetric-modulated arc therapy (VMAT) has advantages over IMRT, the relative merits of HDR-SA brachytherapy vs. VMAT in a more diverse cohort are yet to be established [10–12]. The current study is a dosimetric comparison of the delivered brachytherapy plans to generated VMAT radiation plans for 12 sequential patients who underwent CT-based planning and received HDR-SA treatment between 2011 and 2018.

## 2. Methods

### 2.1. Patient Selection

Twenty patients with face or scalp angiosarcoma were treated with high-dose-rate surface applicator (HDR-SA) brachytherapy in our institution's department of radiation oncology between 2003 and 2018. Radiation plans were available for the 12 patients treated from 2011 to 2018, and these patients formed the basis of the dosimetric comparison between HDR-SA brachytherapy and VMAT. All patients' medical records were reviewed after obtaining approval from our institutional review board.

### 2.2. CT Simulation and Target Volume Delineation

For patients receiving definitive HDR-SA, at the time of CT simulation, radio-opaque wires were used to demarcate the tumor volume and any surgical scars. Radio-opaque wires were also placed adding a 5 cm clinical margin to gross tumor (taking into account anatomic landmarks). To compare the delivered HDR-SA brachytherapy treatment plan to a deliverable VMAT treatment plan, the 100% brachytherapy isodose volume was designated the planning target volume (PTV). Organs at risk (OARs) were delineated on the CT planning scans. Specific OARs included the brain, as well as the bilateral lacrimal glands, orbits, lenses, cochlea, and parotid glands. Of note, two patients did not have the inferiormost aspect of the brain imaged at the time of CT simulation (in the context of the target lesion being located at the apex of the scalp). The prescription dose for all plans was 51 Gy in 17 fractions.

### 2.3. HDR-SA Brachytherapy Treatment Planning

HDR-SA brachytherapy using Iridium-192 was delivered using custom applicators designed to conform to the unique topology of each patient's scalp and/or face. A standard thermoplastic immobilization mask was used to serve as the base for flap applicator attachment. The specific planning technique varied between patients and included inverse planning, manual optimization, or a combination of both. The goal of planning was to deliver 100% of prescription dose to a depth of 3 mm under the skin surface (to reach the dermis) or deeper up to 8 mm to reach the full depth of the tumor. In some cases, it was necessary to generate the plan with a nonconstant depth of the prescription isodose (e.g., 8 mm depth at the center of a target with 3 mm depth at the periphery). While 125% of prescription dose to the skin was generally avoided, in some cases, up to 135% of prescription dose at treatment depths of 3 mm was permitted by the treating attending physician. Homogeneity of dose distribution at a depth of 3 mm was optimized to compensate for possible day-to-day variation in the setup. All treatment plans were initially normalized with respect to the dose points at a depth of 3 mm.

### 2.4. VMAT Treatment Planning

The same prescription was also used for VMAT planning. The VMAT plans were generated on the Eclipse treatment planning system (version 15.6, Varian Medical Systems, Palo Alto, CA), using three coplanar arcs. All plans, except for two, used full 360° arcs to maximize modulation. The partial arc plans had smaller PTV volumes that did not require full arcs. The arcs had combinations of 355°, 5°, 85°, and/or 95° collimator angles to increase modulation ability and to reduce low-dose leakage superiorly and inferiorly. The maximum *X*-jaw length was set to 14.8 cm to allow multileaf collimators (MLCs) to fully close during beam delivery. All plans were designed to be delivered by using a Varian Truebeam linear accelerator equipped with a Millennium 120 MLC. A 5 mm bolus was placed over the PTV to allow for superficial dose, and the PTV was optimized for coverage and conformality using the Photon Optimizer (version 15.6.03) algorithm. OARs were also optimized as needed, but obtaining PTV coverage and conformality were prioritized. The plans were calculated using the Anisotropic Analytical Algorithm (version 13.6.23) with heterogeneity correction. The total MUs per fraction ranged from 700–1400 MU, with a treatment delivery time of approximately 2–4 minutes.

### 2.5. Dosimetric Comparison

The mean dose to each OAR was calculated. For paired OARs (lacrimal glands, orbits, lenses, cochlea, and parotid glands), the mean dose to the left and right for each patient was counted separately. If the entire organ was not included in the CT simulation scan, that particular measurement was not included in the analysis. One patient did not have the right lacrimal gland, right orbit, and right lens fully imaged at CT simulation, and these organs were excluded from the analysis. Thus, with 11 patients having the bilateral visual apparatus included (*n* = 22) and one patient having only the left side of the visual apparatus included (*n* = 1), the total number of visual apparatus organs available for analysis was 23. The same patient did not have either the left or right cochlea imaged, and thus, with 11 patients having the bilateral cochlea included, the total number of cochlea available for analysis was 22. Nine patients had the full bilateral parotid glands imaged, and thus, the total number of parotids available for analysis was 18. Because the entire brain was not included on all CT simulation scans, it was not possible to compare traditional dose volume histogram (DVH) metrics. Instead, the maximum point dose and absolute volume of brain receiving 50% and 80% of prescription dose were compared by the two-tailed nonparametric Mann–Whitney test. Additionally, the brain mean total doses were also compared by the Mann–Whitney test.

## 3. Results

### 3.1. Treatment Context

The primary clinical target was the scalp for 11 patients and the bilateral cheeks for one patient. Two patients had unifocal lesions, five had two sites of disease, and five had multifocal disease. Ten patients received HDR-SA brachytherapy as part of their initial definitive treatment course, most commonly after chemotherapy, while two patients received brachytherapy in the setting of recurrent disease. Three patients underwent surgery prior to radiation therapy. The median number of catheters used for HDR-SA was 25.5 (range 10–69). The number of catheters did not correlate with the anatomic site (e.g., face vs. scalp) but was associated with tumor involving a larger surface area.

### 3.2. Dosimetric Comparison

**Figure 1:**
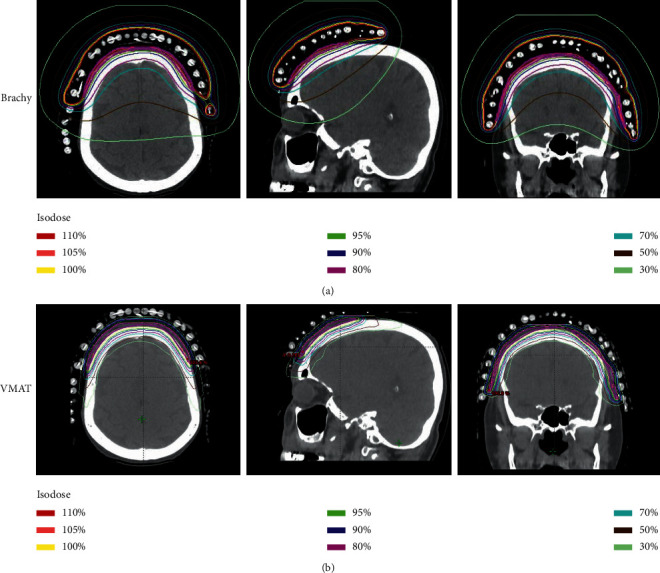
Comparing treatment plans for a single patient. (a) The delivered HDR-SA brachytherapy isodose distribution for a scalp angiosarcoma patient. (b) The paired VMAT isodose distribution for the same patient. Magenta line denotes the PTV.

Deliverable VMAT plans for each patient were created as described and compared to the HDR-SA brachytherapy plans (Figure 1). By definition, for all patients, 100% of the PTV received 100% of the prescription dose by the HDR-SA brachytherapy plan. Each patient's VMAT plan also met our institution's traditional metric for adequate volume coverage with 100% of the PTV receiving at least 95% of the prescription dose (V95% = 100%).

**Figure 2:**
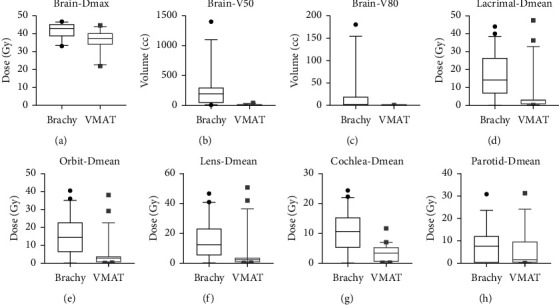
Box-and-whisker plot of dose to organs at risk. (a) Brain point maximum dose. (b) Volume of brain receiving 50% of prescription dose (25.5 Gy). (c) Volume of brain receiving 80% of the prescription dose (40 Gy). (d) Mean lacrimal dose. (e) Mean orbit dose. (f) Mean lens dose. (g) Mean cochlea dose. (h) Mean parotid dose. (box corresponds to 10–90 percentile).

Dose metrics for various organs at risk are shown in Table 1. A significant difference was seen between the HDR-SA brachytherapy and VMAT plans for all parameters studied except mean parotid dose. The median maximum point dose to the brain was 5.5 Gy higher with brachytherapy (Figure 2(a), 43 Gy vs. 37.5 Gy, *p*=0.021) which was similarly reflected in the larger volume of brain receiving 50% of the prescription dose (25.5 Gy) with the brachytherapy plans (Figure 2(b), 194 cc vs. 6.1 cc, *p* < 0.001). While technically the volume of brain receiving 80% of the prescription dose (40 Gy) was also higher with the brachytherapy plans (1.3 cc vs. 0 cc, *p*=0.002), this finding was driven by two outliers whose V80% was 180 cc and 94 cc, respectively (Figure 2(c)).

The median mean orbit, lens, and cochlea doses in the brachytherapy plans ranged between 10 and 15 Gy, while the corresponding mean doses in the VMAT plans ranged between 2 and 4 Gy; all differences were statistically significant (Figures 2(d)–2(g)). There was no significant difference in the mean parotid dose between the VMAT and brachytherapy plans (Figure 2(h)). Patients whose PTV was immediately adjacent to an OAR did not show a dosimetric advantage to VMAT over brachytherapy. Of all the OARs evaluated, the right parotid in a single patient was the only OAR that showed a >5 Gy higher mean dose with VMAT vs. HDR-SA brachytherapy. Conversely, at least half the patients had a >5 Gy higher mean lacrimal, orbit, lens, and cochlea dose with HDR-SA brachytherapy compared to VMAT. The left parotid mean dose was >5 Gy higher with brachytherapy compared to VMAT in 3 patients.

## 4. Discussion

Since 2003, we have treated 20 patients with a multimodality therapy approach including HDR-SA brachytherapy. We had CT planning information for the 12 patients treated since 2011. Our practice has been to prescribe 51 Gy in 17 fractions. This brachytherapy approach was adopted prior to the availability of IMRT or VMAT as it was dosimetrically superior to the then available matched electron photon techniques and 3-D conformal techniques. However, in the context of evolving external beam radiation therapy techniques, we sought to compare dosimetric coverage and conformality of the modern VMAT technique vs. HDR-SA brachytherapy.

This study is the first to dosimetrically compare HDR-SA brachytherapy to VMAT. For the patients in our cohort, we found that VMAT confers significant dosimetric advantages to almost all OARs for the majority of patients which suggests that HDR-SA brachytherapy may not be the optimal default treatment approach, but rather best utilized for only a subset of patients based on anatomic considerations. The most consistent advantage for VMAT over HDR-SA brachytherapy was the reduction of the brain dose delivered. All 12 patients treated with brachytherapy had a higher brain point max dose (median 43 Gy vs. 37.5 Gy), as well as volume of brain receiving 50% of prescription (median 194 cc vs. 6.1 cc). In addition, no VMAT plan had >1 cc of brain receiving 80% of the prescription dose, while half of the HDR-SA brachytherapy plans crossed this threshold. While the clinical significance of these differences is not well established, given recent data showing frequent neurocognitive deficits in head and neck cancer patients receiving radiation, limiting radiation dose to the brain is likely beneficial [14].

The mean radiation dose to the structures of the eye and the cochlea were generally quite low with VMAT (median 2–4 Gy). The HDR-SA brachytherapy mean dose to these OARs was at least 5 Gy higher in half or more patients. If one assumes that the *α*/*β* of normal tissue is 2-3, the traditional EQD2 26 Gy mean dose constraint of the lacrimal gland was exceeded in the HDR-SA brachytherapy plans for five lacrimal glands (belonging to three patients), while only two lacrimal glands (belonging to one patient) were exceeded in the VMAT plans, suggesting dry eye might be more common after brachytherapy treatment. Similarly, an EQD2 10 Gy mean lens dose constraint was exceeded in the HDR-SA brachytherapy plans for 12 lenses (belonging to six patients), while only three lenses (belonging to two patients) exceeded this metric in the VMAT plans, suggesting cataracts might be more common after brachytherapy treatment. There were no cases where the traditional orbit or cochlea dose constraints were exceeded in either the HDR-SA brachytherapy or VMAT plans.

An important caveat of these findings is that, in cases where the PTV was immediately adjacent to an OAR, significant sparing while ensuring target volume coverage was not possible in either the HDR-SA brachytherapy or VMAT plans. In such cases, the dosimetric advantages of VMAT for structures of the eye or cochlea are less apparent. In addition, depending on patient anatomy and placement of bolus, a theoretical VMAT plan may not always be reproducibly deliverable. In these cases, HDR-SA brachytherapy or electrons may be preferred.

Limitations of this study include the fact that target volumes are not routinely contoured prior to HDR-SA brachytherapy. As a result, to compare clinical target coverage between the two techniques, we biased the comparison in favor of HDR-SA brachytherapy by designating the VMAT PTV as the volume covered by the 100% prescription isodose line of the HDR-SA brachytherapy plan. In reality, this may not have been precisely the same as the disease target. Importantly, we were unable to compare efficacy of VMAT vs. HDR-SA brachytherapy though would assume similar disease control rates in the context of similar coverage of the target volume. However, despite these limitations, we believe this study provides important information regarding the possible dosimetric advantages to a VMAT approach.

While our institutional practice has been to use HDR-SA brachytherapy as the radiation modality of choice, the results of this study suggest VMAT is likely to confer a dosimetric advantage for many patients. Despite this finding, individual patient factors such as tumor location, anatomy, and reproducibility of bolus placement may continue to make HDR-SA brachytherapy the appropriate technique for a subset of patients. Going forward, we plan to compare both techniques for each patient as, ultimately, a personalized approach is likely to be the optimal treatment plan.

## Figures and Tables

**Table 1:** Dosimetric comparison of HDR-SA brachytherapy and VMAT for organs at risk.

	*n* ^a^	Median brachy^b^	Median VMAT^c^	*p* value

Brain Dmax	12	43 Gy	37.5 Gy	0.021
Brain V50% (25.5 Gy)	12	194.0 cc	6.1 cc	<0.001
Brain V80% (40 Gy)	12	1.3 cc	0.0 cc	0.002
Mean lacrimal gland	23	14.2 Gy	2.9 Gy	0.007
Mean orbit	23	14.5 Gy	2.8 Gy	0.004
Mean lens	23	12.3 Gy	2.4 Gy	0.014
Mean cochlea	22	10.6 Gy	3.4 Gy	<0.001
Mean parotid	18	16.9 Gy	4.3 Gy	0.989

Key: ^a^*n*: number, ^b^brachy: brachytherapy, ^c^VMAT: volumetric-modulated arc therapy.

## Data Availability

The electronic medical record data used to support the findings of this study are restricted by the Dana-Farber Cancer Institute (DFCI) IRB in order to protect patient privacy. Data are available from Miranda B. Lam, miranda_lam@dfci.harvard.edu, for researchers who meet the criteria for access to confidential data.
